# Ginsenoside Rg3 Ameliorates Psoriasis‐Like Dermatitis through Inhibition of NF‐κB/NLRP3 Inflammasome Signaling and Regulating Th17/Treg Balance

**DOI:** 10.1002/iid3.70362

**Published:** 2026-02-16

**Authors:** Liyun Gao, Qingge Xie, Zhaoli Zhou, Wanlu Zhang, Huiya Sun, Zhen Yue, Congjun Jiang

**Affiliations:** ^1^ Anhui Key Laboratory of Tissue Transplantation Bengbu Medical University Bengbu Anhui China; ^2^ Department of Dermatology First Affiliated Hospital of Bengbu Medical University Bengbu Anhui China; ^3^ School of Laboratory Bengbu Medical University Bengbu Anhui China

**Keywords:** Ginsenoside Rg3, NF‐κB, NLRP3, psoriasis, Th17/Treg

## Abstract

**Background:**

Psoriasis, characterized as a persistent cutaneous inflammatory condition driven by aberrant immunity, exhibits pathogenesis and disease course heavily influenced by sustained inflammatory activity. Ginsenoside Rg3 (Grg3), a traditional Chinese medicinal compound, has shown notable therapeutic effects on various inflammatory diseases. However, its therapeutic efficacy and mechanisms of action in the treatment of psoriasis remain unclear.

**Method:**

In this study, we utilized 6 to 8‐week‐old female BALB/c mice and applied 5% imiquimod (IMQ) cream to their dorsal skin to establish a psoriasis‐like dermatitis model. Twenty‐five mice were randomly divided into five groups: control, model, and the Grg3‐L/M/H groups, receiving dosages of 5, 10, and 20 mg/kg/day, respectively, with three mice in each group. Grg3 was administered continuously for 7 days to evaluate its effects on the skin of the psoriasis mice. The following methodologies were employed: the Psoriasis Area and Severity Index) for clinical severity scoring, hematoxylin‐eosin staining for histomorphological analysis, alongside immunohistochemistry, immunofluorescence, and flow cytometry for detailed protein and cellular profiling to assess the expression levels of the keratinocyte proliferation marker Ki‐67, inflammatory cytokines, NLRP3 inflammasome‐related factors, and NF‐κB pathway‐related proteins in vivo.

**Result:**

Significant reductions in PASI scores were observed in IMQ‐treated BALB/c murine models of psoriasis following Grg3 treatment. It decreases epidermal thickness, inhibits the proliferation and differentiation of epidermal cells, and reduces the expression of the inflammatory cytokine interleukin‐17 (IL‐17) in splenic lymphocytes. Additionally, an increase in Foxp3 + CD4+ regulatory T cell (Treg) proportion and a decrease in the expression levels of NLRP3, apoptosis‐associated speck‐like protein (ASC), caspase‐1, and IL‐1β were observed following Grg3 administration. Furthermore, A concomitant downregulation of p‐p65 expression and its downstream effectors, such as the pro‐inflammatory cytokines interleukin‐6 (IL‐6) and tumor necrosis factor‐alpha (TNF‐α), was also observed.

**Conclusion:**

Our findings indicate that Grg3 confers protective effects in a murine model of imiquimod‐induced psoriasis‐like dermatitis. The potential therapeutic properties of Grg3 potentially involve modulation of NLRP3 inflammasome activation, suppression of NF‐κB signaling, and restoration of Th17/Treg cell homeostasis.

## 1 Introduction

Psoriasis is a common, chronic inflammatory skin disorder with significant global prevalence, driven fundamentally by dysregulation of the immune system, generally believed to be influenced by a combination of genetic, immune, and environmental factors. It can manifest on any part of the skin, but it most commonly affects areas such as the forearms, the skin behind the ears, and the scalp [1]. It is reported that the global incidence of psoriasis accounts for approximately 2%–3% [2]. In psoriasis, abnormal epidermal thickening ensues due to uncontrolled proliferation and aberrant differentiation of keratinocytes. The primary manifestations include red patches covered with silvery scales [3]. In addition to skin‐related concerns, Psoriatic patients face a significantly increased risk for developing depression and exhibit an increased incidence of cardiovascular diseases, diabetes, and arthritis compared to the general population. The primary manifestations and complications of psoriasis significantly impact the physical and mental health, as well as the socioeconomic status, of affected individuals [4]. Therefore, exploring the pathogenesis of psoriasis and identifying suitable therapeutic drugs for psoriasis have become the primary tasks.

The intricate mechanisms responsible for psoriasis development remain incompletely defined. Nevertheless, it is established that aberrant immune cell activity and inflammatory signaling significantly fuel both the initiation and perpetuation of this disorder [5]. Numerous studies have demonstrated that the incidence of psoriasis is predominantly linked to the aberrant expression of various inflammatory factors produced by immune cells, including TNF‐α, IL‐1β, IL‐6, and IL‐17A [6, 7]. These inflammatory factors stimulate and activate IL‐23‐producing myeloid dendritic cells, which increases the production of IL‐17 inflammatory factors in TH17 cells. This process subsequently activates keratinocytes, promoting the aggregation of inflammatory cells and resulting in an inflammatory response [8]. Regulatory T (Treg) cells represent a specialized subset of helper T cells that play a critical role in the regulation of autoimmunity. Accumulating evidence establishes Treg cells as critical regulators of immune homeostasis. Moreover, dysregulation of the Th17/Treg cell ratio critically contributes to psoriasis pathogenesis [9].

Numerous signaling pathways are closely associated with the onset and progression of psoriasis. A critical involvement of Toll‐like receptors (TLRs) in psoriasis etiology is supported by prior investigations. Notably, IMQ, functioning as a TLR agonist, induces psoriasis‐like disease in mice. Upon activation by IMQ, TLR7/8 stimulates the NF‐κB cascade, which orchestrates the upregulation of diverse inflammatory cytokines and drives the IL‐23/Th17 inflammatory pathway. Interestingly, certain inflammatory cytokines, such as TNF‐α, IL‐17, and IL‐23, depend on NF‐κB as their downstream mediator, creating a positive feedback loop that further promotes the initiation and progression of inflammation [10]. In recent years, the NLRP3 inflammasome has attracted significant attention for its involvement in the onset and progression of psoriasis. The core constituents of this complex include NLRP3, caspase‐1 (cysteine‐aspartic acid‐specific protease 1), and ASC—an apoptosis‐associated speck‐like adaptor protein harboring a caspase recruitment domain (CARD) [11]. Studies have demonstrated that various risk factors can enhance the production of the inflammatory cytokine IL‐1β via caspase‐1 activation in the NLRP3 inflammasome, which subsequently activates immune cells. This process further stimulates the release of inflammatory cytokines, including INF‐γ and TNF‐α, thereby contributing to the onset and progression of psoriasis [12]. Recent investigations have increasingly focused on the NLRP3 inflammasome's contribution to psoriasis. Notably, this inflammasome complex exhibits significant upregulation within the stratum corneum of murine psoriasiform lesions induced by IMQ. Furthermore, in vivo studies utilizing psoriasis models reveal pronounced enhancement of NLRP3 inflammasome expression relative to normal tissues [13]. Studies have also shown that inhibitors related to the NLRP3 inflammasome can ameliorate the associated inflammation in psoriasis‐like mouse [14]. Various studies indicate that this inflammasome may serve as a novel therapeutic target for psoriasis, with evidence suggesting a close relationship between the NF‐κB and NLRP3 inflammasome pathways.

Currently, several treatments for psoriasis are associated with numerous side effects. In recent years, traditional Chinese medicines known for their anti‐inflammatory properties have gained popularity in clinical practice. Grg3, a bioactive component derived from ginseng, has substantial evidence supporting its diverse therapeutic potentials. However, the role and mechanism of Grg3 in psoriasis remain unknown. Given that the NF‐κB and NLRP3 signaling pathways are the core drivers of psoriasis inflammation, and the Th17/Treg immune imbalance is a key feature, we speculate that Grg3 may restore the Th17/Treg balance by inhibiting the NF‐κB/NLRP3 axis, thereby alleviating psoriasis. To verify this hypothesis, this study aims to evaluate the therapeutic effect of Grg3 in an IMQ‐induced psoriasis‐like mouse model, explore its regulatory role in the NF‐κB and NLRP3 pathways, and clarify its influence on the differentiation of Th17/Treg cells.

## 2 Materials and Methods

### 2.1 Laboratory Animals and Reagents

Ginsenoside Rg3 (CAS Registry Number: 14197‐60‐5) with a purity of above 99% was purchased from MedChemExpress Company. Female BALB/c mouse, aged 6–8 weeks, were procured from Jiangsu Qinglongshan Biotechnology Co. Ltd. The housing conditions were standardized with a daily lighting schedule, and laboratory settings were regulated to 25 ± 2°C ambient temperature and 55 ± 5% relative humidity. The mouse had unrestricted access to tap water and were provided with a nutritionally balanced diet. Prior to the experiment, the mice were acclimatized to their environment of cage of animals and housed for 1 week. All protocols for in vivo experiments were performed according to the NIH Guide for the Care and Use of Laboratory Animals. Approval for all procedures involving animals was obtained from the Institutional Animal Care and Use Committee (IACUC) at the First Affiliated Hospital of Bengbu Medical University (Approval no. 2024618).

### 2.2 Establishment of Psoriasis Models

Mouse (*n* = 15) underwent random allocation into five distinct groups: the control group, the model group, the Grg3‐Low group, the Grg3‐Middle group, and the Grg3‐High group. In the control group, after a 7‐day acclimatization period, the hair on the dorsal skin of the mice was removed to expose an area of approximately 3 cm × 4 cm, and the mice were subsequently fed normally without any further treatment. In the model group, following the same acclimatization period, following dorsal hair removal, 62.5 mg of 5% IMQ cream was topically administered daily to the shaved area for seven successive days to induce psoriasis‐like pathology. For the Grg3‐L, Grg3‐M, and Grg3‐H groups, based on the IMQ model, Grg3 was administered via oral gavage for 7 consecutive days at doses of 5, 10, and 20 mg/kg [15], respectively. We anesthetized all mouse on Day 8 through intraperitoneal injection of 1% sodium pentobarbital (30 mg/kg), followed by euthanasia via cervical dislocation. All procedures adhered to animal ethics and complied with the guidelines set forth by Bengbu Medical University.

### 2.3 Psoriasis Area and Severity Index (PASI) Score and Phenotypic Observation in Mouse

The severity of psoriasis on the dorsal skin of the mouse was assessed macroscopically by evaluating the scales, erythema, and hypertrophy, with PASI scores assigned accordingly. The evaluation of psoriasis severity was based on three criteria: erythema, scales, and thickness. The scoring system utilized was as follows: 0 ‐ none; 1 ‐ mild; 2 ‐ moderate; 3 ‐ marked; 4 ‐ very marked. The researchers (Liyun Gao) responsible for providing intervention measures to patients and the personnel (Qinge Xie) responsible for outcome evaluation are completely independent teams. The assessor (Qinge Xie) evaluated the condition and PASI score of the patients at baseline and during follow‐up without knowing the grouping information of the patients. All image data are anonymized and then analyzed by image analysts.

### 2.4 Hematoxylin‐Eosin (HE) Staining

After the skin tissues from each group of mice were excised and fixed, they were subsequently embedded and sectioned. The sections underwent deparaffinization by treatment with xylene for 5 to 10 min, followed by two washes in 100% ethanol. They were then hydrated sequentially in 95%, 85%, and 75% ethanol, with each step lasting 30 s. Tissue sections underwent Hematoxylin staining for 15 to 30 min, followed by a 1 to 3 min wash under running water, and subsequently stained with Eosin for approximately 1 to 3 min, followed by another rinse with running water. The sections were rapidly dehydrated by passing through 100% ethanol and xylene, then mounted on slides using neutral resin and covered with coverslips. Ultimately, microscopic examination of the sections was conducted, and digital images were acquired.

### 2.5 Immunohistochemistry

Following the euthanasia of the mice, the dorsal skin was promptly excised and fixed in paraformaldehyde (PFA). The skin tissue underwent dehydration through a graded series of ethanol, after which it was embedded in paraffin. Upon cooling and solidification, the tissue was sectioned into slices of 5–10 μm thickness and mounted onto pre‐treated slides. Antigen retrieval was conducted using a sodium citrate buffer in either a microwave or water bath. After washing the sections, to mitigate non‐specific binding, tissue sections were blocked using a 1% bovine serum albumin (BSA) buffer and incubated at ambient temperature for 60 min. Primary antibodies targeting Ki67, NLRP3, IL‐1β, Caspase‐1, and ASC were then applied, typically with overnight incubation at 4°C. On the subsequent day, following PBS washes, diluted secondary antibodies were added and incubated at room temperature for an additional hour. For antibodies labeled with peroxidase, a subsequent 3,3'‐diaminobenzidine (DAB) chromogenic reaction was performed, followed by documentation of the staining results.

### 2.6 Immunofluorescence

Skin tissue from mouse was fixed in paraformaldehyde. The fixed tissue was then processed into embedding blocks, sectioned using a microtome, and mounted on glass slides. The sections were treated with PBS solution containing 5% BSA and blocked at room temperature for 1 h. Specific primary antibodies against IL‐6, TNF‐α, and P‐P65 were selected, diluted according to the manufacturer's instructions, and applied to the sections, after overnight 4°C incubation and PBS washing, fluorochrome‐conjugated secondary antibodies (FITC/TRITC) were incubated at room temperature for 1 h. Nuclear visualization was achieved through DAPI counterstaining (10 min), followed by microscopic examination.

### 2.7 Flow Cytometry

Spleens harvested from the respective mouse cohorts underwent dissection to eliminate adjacent extraneous tissue. Subsequently, the tissue was mechanically dissociated, enzymatically digested, and sequentially filtered (70‐μm mesh) and centrifuged to generate single‐cell suspensions. These cells were then stimulated with phorbol 12‐myristate 13‐acetate (PMA), ionomycin, and brefeldin A (BFA), cells were incubated under standard conditions (37°C, 5% CO₂ humidified atmosphere). For immunophenotyping, cells were stained with the following fluorescein‐conjugated monoclonal antibodies: CD4‐FITC, CD3‐APC, CD25‐APC, and an IL‐17‐specific antibody (all sourced from Cell Signaling Technology), using a room temperature incubation in the dark. Following fixation and permeabilization for nuclear antigens, an intracellular antibody targeting FOXP3‐APC was applied. Flow cytometric data acquisition concluded the experimental procedure.

### 2.8 Data Analysis

Statistical analyses were performed utilizing SPSS software (version 22.0). Data, derived from a minimum of three separate experimental replicates, are expressed as the arithmetic mean ± SD. To evaluate inter‐group differences across multiple conditions for the specified parameters, Data were analyzed using one‐way ANOVA, and Tukey's test was used for post hoc pairwise comparisons. with statistical significance set at *p* < 0.05.

## 3 Result

### 3.1 Grg3 Treatment Ameliorates Imiquimod‐Induced Psoriasis‐Like Skin Lesions

**FIGURE 1:**
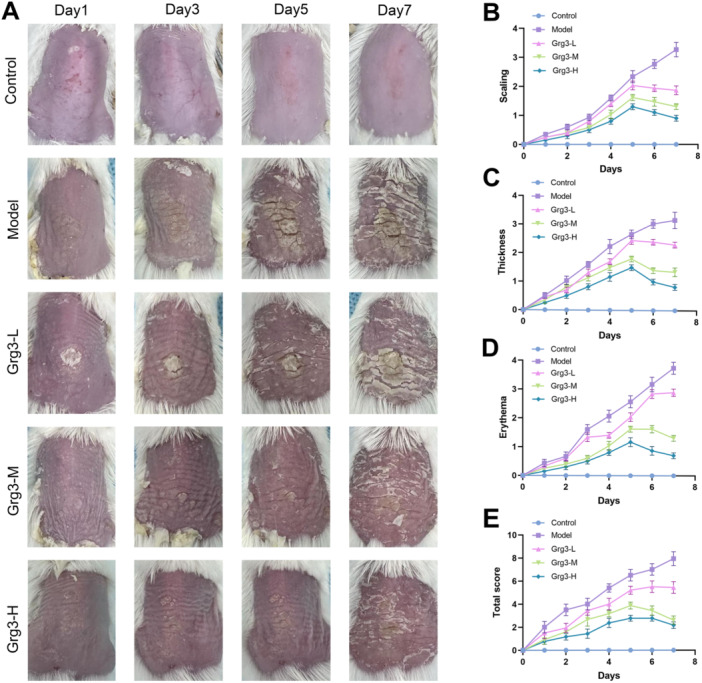
illustrates the effects of Ginsenoside Rg3 (Grg3) on the morphological and histological characteristics of imiquimod (IMQ)‐induced psoriasiform dermatitis in mice. (A) This panel presents representative gross views of the dorsal skin of BABL/C mice following 7 consecutive days of treatment. (B–E) Daily assessments were conducted to evaluate scaling, thickness, and erythema of the dorsal skin, utilizing a scoring system ranging from 0 to 4. Furthermore, the cumulative score, calculated as the sum of scaling, thickness, and erythema, is also presented.

To investigate the therapeutic effects of Grg3 on IMQ‐induced psoriasis‐like mice, the Grg3 treatment group was administered Grg3 via gavage for seven consecutive days following the modeling phase. The PASI was employed to quantify the clinical severity of skin lesions, characterized by features such as scaling, erythema, and thickening of the skin on the back. The control group exhibited smooth skin without significant lesions, while the IMQ‐induced model group demonstrated pronounced scaling, erythema, and skin thickening, as illustrated in Figure 1A. As anticipated, the skin lesions in the Grg3‐treated group exhibited a clear dose‐dependent alleviation of symptoms. Compared to the model group, the Grg3‐treated group showed significant improvements in scaling, erythema, and skin thickening, accompanied by a reduction in PASI scores, as depicted in Figure 1B–E. These findings indicate that Grg3 has a substantial ameliorative effect on the skin symptoms of IMQ‐induced psoriasis‐like mouse.

### 3.2 Grg3 Treatment Modifies the Histological Characteristics of Imiquimod (IMQ)‐Induced Dorsal Skin in Mouse and Inhibits the Proliferation and Differentiation of Epidermal Cells

**FIGURE 2:**
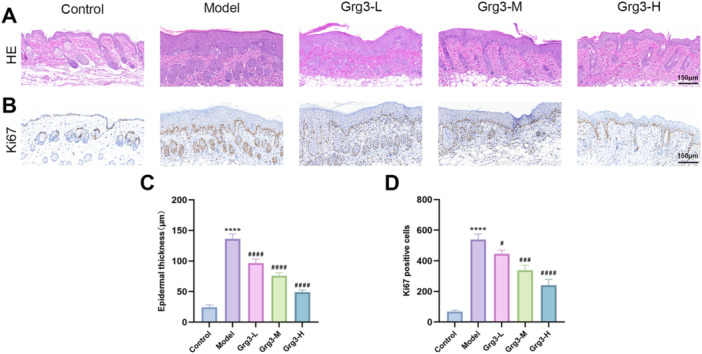
illustrates that Grg3 can modify the histological characteristics of the mice model of psoriasis and inhibit Figure 2: the proliferation and differentiation of epidermal cells. (A) The back skin of mice with IMQ‐induced psoriasis‐like lesions was assessed using histological techniques, specifically staining with hematoxylin and eosin (H&E) at a magnification of 200×, with a scale bar representing 150 µm. (B) The expression of Ki‐67 in the skin of each group of mice was determined through immunohistochemistry (200×). (C) The epidermal thickness of the back skin was measured on Day 8. (D) The number of positive cells in the basal layer of the skin was quantified on Day 8. The data are expressed as the mean ± SD of three independent experiments. *compared with the control group: ****p < 0.0001; ^#^comparisons with the model group: ^#^ *p* < 0.05, ^###^ *p* < 0.001, and ^####^ *p* < 0.0001. IMQ, Imiquimod.

The pathological changes in skin tissue induced by IMQ were evaluated using hematoxylin and eosin (H&E) staining. As illustrated in Figure 2A, the IMQ‐induced model group exhibited pathological psoriatic lesions, which included a significantly thickened epidermis, infiltration of inflammatory cells in the superficial layer of the epidermis, thinning and disappearance of the granular layer, acanthosis, and downward extension of the epidermal projections. The disease induction group displayed substantially greater epidermal thickness than the control group. Conversely, the epidermal thickness in the low, medium, and high‐dose gavage treatment groups demonstrated a dose‐dependent thinning, as depicted in Figure 2C.

Ki‐67 is a marker protein involved in the proliferation of keratinocytes. To investigate the therapeutic effects of Grg3 on psoriasis and its potential association with the proliferation of skin keratinocytes, this study employed immunohistochemical methods to detect the expression of Ki‐67 in mouse skin. Quantitative results from the immunohistochemical analysis indicated that, compared to the control group, the psoriasis lesions in the model group exhibited a significant increase in the proliferation of skin keratinocytes, as illustrated in Figure 2B. In contrast, the proliferation of keratinocytes in the psoriasis lesions of the three groups of mice treated with Grg3 demonstrated a decreasing trend, as depicted in Figure 2D. These results suggest that treatment with Grg3 effectively reduced the abnormal proliferation of keratinocytes in IMQ‐induced psoriasis lesions.

### 3.3 Grg3 Treatment Inhibits the IMQ‐Activated NLRP3 Inflammatory Signaling Pathway

**FIGURE 3:**
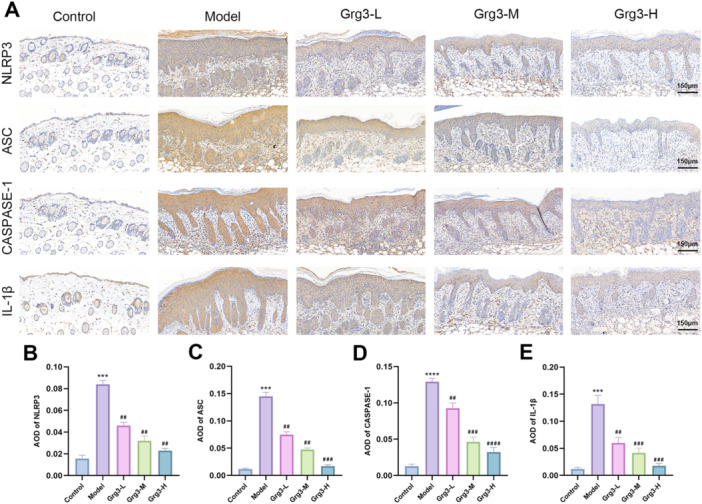
Grg3 suppresses the nucleotide‐binding oligomerization domain (Nod)‐like receptor family pyrin domain containing 3 (NLRP3) inflammasome signaling activated by IMQ. (A) Expression of NLRP3, ASC, caspase‐1, and IL‐1β was analyzed via immunohistochemistry at 200× magnification. (B–E) The optical density values (AOD) for NLRP3, ASC, caspase‐1, and IL‐1β are presented. The data are expressed as the mean ± SD of three independent experiments. Statistical comparisons indicate that *compared to the control group: ***p < 0.001 and ****p < 0.0001; ^#^comparisons with the model group: ^#^ *p* < 0.01, ^##^ *p* < 0.001, and ^####^ *p* < 0.0001. IMQ, Imiquimod.

Studies have demonstrated a close correlation between the activation of the NLRP3 inflammasome and the expression of inflammatory mediators. Consequently, this study utilized immunohistochemical methods to assess NLRP3 inflammasome expression. The results revealed a significant increase in NLRP3 expression in the skin tissues of mice from the IMQ‐induced model group compared to the control group. Moreover, elevated expression levels were detected for ASC, caspase‐1, and IL‐1β, while the expression of various factors in the skin of mice treated with Grg3 exhibited a dose‐dependent decrease, as depicted in Figure 3A. Notably, IL‐1β, a pro‐inflammatory factor, may play a role in the skin inflammation process, with its expression significantly reduced in the skin of Grg3‐treated mouse, as illustrated in Figure 3E.

### 3.4 Grg3 Treatment Enhances the Therapeutic Effect on Psoriasis by Inhibiting the NF‐κB Signaling Pathway

**FIGURE 4:**
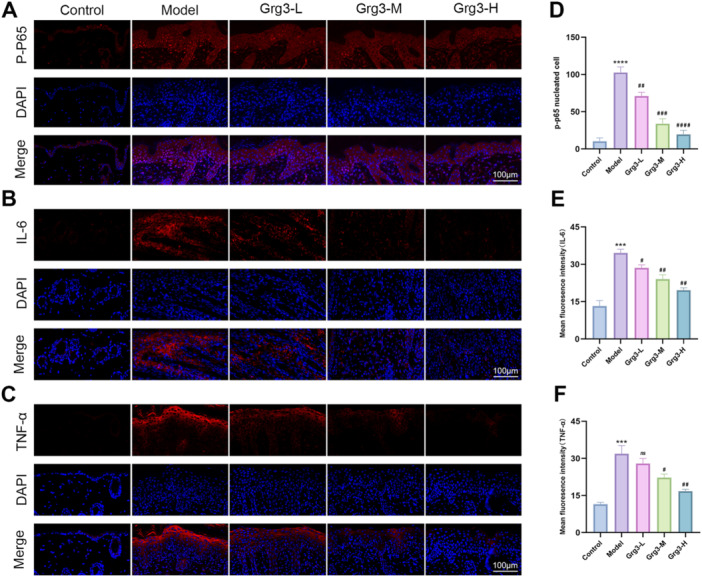
Grg3 inhibits the activation of the NF‐κB signaling pathway. (A–C) The expression of P‐P65, IL‐6, and TNF‐α proteins was detected by immunofluorescence (500×). (D) The number of nuclear P‐P65‐positive cells in mouse skin tissue. (E, F) The mean fluorescence intensity of IL‐6 and TNF‐α in mouse skin tissue. The data are expressed as the mean ± SD of three independent experiments. *Compared with the control group: ***p < 0.001; ^#^compared with the model group: ^#^ *p* < 0.05, ^##^ *p* < 0.01, ^###^ *p* < 0.001, ^####^ *p* < 0.0001.

The NF‐κB signaling cascade has been demonstrated to play an essential role in inflammatory processes, according to prior investigations. Therefore, we employed immunofluorescence staining on mouse skin to investigate the expression of NF‐κB‐related signaling factors and downstream inflammatory mediators, as illustrated in Figure 4. In the control group, the expression levels of P‐P65, TNF‐α, and IL‐6 were nearly undetectable in the mouse skin. In contrast to the control group, the skin of mice stimulated with IMQ exhibited significant nuclear translocation of P‐P65, while TNF‐α and IL‐6 showed markedly elevated fluorescence signal expression, as depicted in Figure 4A–C. Conversely, Grg3‐treated mice displayed a significant reduction in the expression of these fluorescence signals in the skin, as illustrated in Figure 4D,E. These results suggest that Grg3 may exert a protective effect against IMQ‐induced psoriasis, potentially through the inhibition of NF‐κB pathway activation.

### 3.5 Grg3 Treatment Affects the Ratio of IL‐17 Inflammatory Factor and FOXP3 Cytokine in the Spleen of Mouse

**FIGURE 5:**
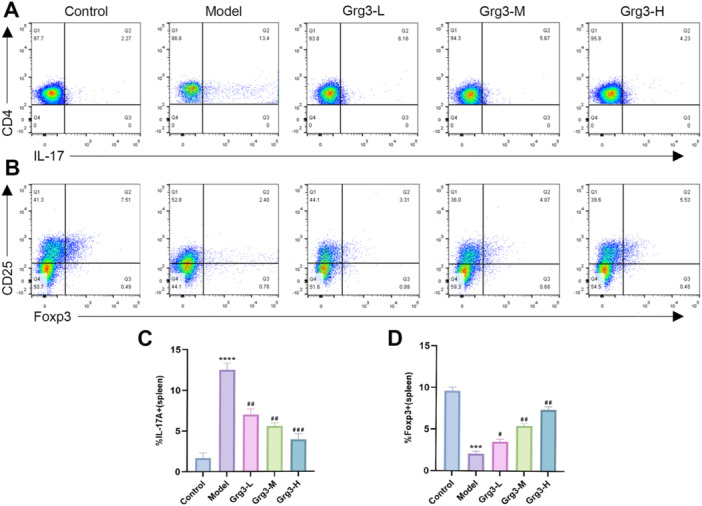
Grg3 inhibits the expression of the IL‐17 inflammatory factor and enhances the proportion of FOXP3 cytokine in mouse splenic cells. (A, B) Flow cytometry was used to detect the expression of IL‐17 and FOXP3 in mouse spleen. (C, D) The proportions of IL‐17 and FOXP3 in mouse splenic cells, with values expressed as the mean ± SD of three independent experiments. *indicates comparison with the control group: ***p < 0.001, ****p < 0.0001, # indicates comparison with the model group: #*p* < 0.05, ##*p* < 0.01, ###*p* < 0.001, ####*p* < 0.0001.

It has been established that IL‐17 is a crucial inflammatory factor in the pathogenesis of psoriasis. Recent studies have indicated that CD4+Foxp3+ Treg cells significantly contribute to the development of psoriasis‐related inflammation. In this study, we employed flow cytometry to examine the expression levels of the IL‐17 inflammatory factor and the FOXP3 cytokine in the spleens of mouse. The results, illustrated in Figure 5A,B, revealed that the control group exhibited no significant expression of the IL‐17 inflammatory factor, while the expression of the FOXP3 cytokine was notably increased. In contrast, the model group demonstrated substantial expression of the IL‐17 inflammatory factor and a marked decrease in FOXP3 cytokine expression in the spleens of the mouse. Mouse treated with Grg3 showed dose‐dependent decreases in IL‐17 expression and corresponding increases in FOXP3 expression. These findings suggest that Grg3 treatment may potentially ameliorate the Th17/Treg immune imbalance.

## 4 Discussion

Psoriasis represents a common chronic inflammatory dermatosis, defined principally by epidermal hyperproliferation and impaired skin barrier integrity [16]. Evidence suggests that psoriatic pathogenesis involves a complex interplay of epidermal and dermal immune responses, coupled with inflammatory cell infiltration into the skin. This process subsequently leads to increased keratinocyte proliferation and abnormal differentiation, thereby activating the immune response. Moreover, the expression of several inflammatory mediators in the skin, such as IL‐17 and IL‐23, further enhances the immune response [17]. Recent studies have revealed that the limitations of certain topical medications for psoriasis have hindered their use and development. Consequently, identifying suitable drug treatments for psoriasis has emerged as a significant area of research in recent years [18]. Research on natural products has increasingly garnered attention. Ginsenosides, which are natural active compounds, primarily consist of RG1, RB1, CK, and RG3. Evidence from research indicates that Grg3 exhibits anti‐inflammatory and immunomodulatory activities [19]. However, the role of Grg3 in psoriasis has rarely been studied.

IMQ is a well‐known and effective immune activator. The application of IMQ to the ears or backs of mice induces erythema, scaling, thickening, and proliferation of epidermal keratinocytes, as well as an increased expression of related inflammatory cytokines in the body. These phenomena align with the manifestations of psoriasis [20]. The IMQ‐induced mouse model of psoriasis is widely utilized in research concerning both the treatment and mechanisms underlying psoriasis. In our study, the topical application of IMQ on the backs of BABL/C mouse successfully induced psoriasis‐like skin symptoms that are consistent with those reported in existing literature. Treatment with Grg3 significantly alleviated epidermal thickening and reduced the Psoriasis Area and Severity Index (PASI) score in the mouse. Activated keratinocytes play a crucial role in the pathogenesis and progression of psoriasis. Our research further demonstrated that Grg3 markedly inhibited the expression of the keratinocyte differentiation marker Ki67 and mitigated epidermal thickening in the mouse. Additionally, activated keratinocytes can increase the expression of inflammatory cytokines, thereby promoting the further development of psoriasis.

It is well established that, in addition to skin lesions, IMQ‐induced psoriasis can also present with systemic inflammatory responses, including splenomegaly, lymphadenopathy, and changes in T and B lymphocyte populations. The spleen, as a vital immune organ, has the capacity to generate various immune cells. Interleukin‐17 (IL‐17), a pivotal pro‐inflammatory cytokine secreted by effector T cells, plays a significant role in the chronic inflammatory processes associated with psoriasis [21]. Studies have demonstrated that FOXP3+ Treg cells can reflect the activation capacity of regulatory T cells (Tregs), suggesting that the regulation of the Th17/Treg balance may ameliorate immune dysregulation in patients with psoriasis. Consequently, targeting the Th17/Treg equilibrium has become a central strategy for elucidating psoriatic pathogenesis and advancing targeted therapeutic development [22]. Additionally, studies have demonstrated that in normal mice and humans, Th17 and Treg cells maintain a state of equilibrium. In contrast, psoriatic mice exhibit an immune imbalance characterized by a decrease in FOXP3 expression and an opposing increase in IL‐17 levels. The function of FOXP3‐positive cells is frequently suppressed, resulting in a dysregulation of the immune system [23]. Simultaneously, the genesis of inflammatory skin lesions has been linked by studies to a failure in negatively regulating NF‐κB signaling in epithelial cells and T lymphocytes [24]. Our study also demonstrated an imbalance in the Th17/Treg cell ratio in IMQ‐induced psoriatic mice. However, following Grg3 treatment, the proportion of FOXP3+ cells exhibited a dose‐dependent increase, while the pro‐inflammatory cytokine IL‐17 was significantly reduced, aligning with findings from previous studies. Our research confirmed that Grg3 influences T cells in the spleen of psoriatic mice; however, additional evidence is required to ascertain whether it exerts a broader impact on systemic immune cells. Future studies specifically designed to measure systemic cytokine levels and distant organ histology are necessary to confirm this hypothesis. These results provide preliminary evidence that Grg3 may alleviate psoriatic‐like skin lesions by modulating the balance of Th17/Treg cells. Additionally, it is worth further exploring whether the activation of Treg cells is closely associated with the NF‐κB pathway.

Functioning as a key transcriptional controller, NF‐κB typically adopts an inactive cytoplasmic p50/p65 heterodimeric form. The ensuing signaling cascade is essential for inflammatory regulation and substantially underpins psoriasis pathogenesis. Upon exposure to various stimuli, such as bacteria, viruses, and cytokines, Nuclear translocation of activated NF‐κB enables its binding to cognate gene promoters and transcriptional induction, thereby facilitating widespread participation in the control of immune and inflammatory signaling [25]. IL‐6 and TNF‐α are the principal inflammatory cytokines activated by the NF‐κB signaling pathway, and these cytokines play crucial roles in mediating the inflammatory response [26]. The simultaneous release of TNF‐α activates a positive feedback loop that further stimulates NF‐κB, thereby exacerbating inflammation. This finding indicates that NF‐κB plays a significant role in the cascade of inflammatory diseases. Research has established that NF‐κB promotes the pathogenesis of psoriasis through the upregulation of MyD88 and p‐NF‐κB expression [27]. In addition, Grg3 can inhibit oxidative stress and myocardial hypertrophy by attenuating the activation of NF‐κB [28]. Another active form of ginsenoside RG1 exerts therapeutic effects on psoriasis by influencing the phosphorylation of IκB kinase α (IKKα) and promoting the nuclear translocation of P65 [29]. In our study, we observed that Grg3 treatment influenced the nuclear translocation associated with the NF‐κB pathway. Furthermore, we demonstrated the activation of this pathway by examining the upstream and downstream inflammatory factors, specifically IL‐6 and TNF‐α, that modulate it. The fluorescence intensity of IL‐6 and TNF‐α in the skin of psoriasis mice treated with Grg3 was significantly reduced. However, the TNF‐α fluorescence value of the Grg3‐L group did not show statistical significance when compared with that of the model group mouse. We considered whether this was related to the relatively small sample size. At the same time, unfortunately, we did not further investigate the underlying molecular mechanisms of NF‐κB activation or whether the inflammatory factors might impact other pathways. Our study provides preliminary evidence suggesting that Grg3 may promote the onset and progression of inflammation by affecting NF‐κB activation.

The NLRP3 inflammasome, a member of the NOD receptor family, is expressed in various cell types, including neutrophils, keratinocytes, and epithelial cells. Its activation has been shown to be closely linked to numerous inflammatory diseases and serves as a pivotal regulator of pro‐inflammatory cytokine production [30]. NLRP3 detects pathogen‐associated molecular patterns and assembles into an active complex alongside the adaptor ASC and the cysteine protease caspase‐1. This interaction facilitates the cleavage of pro‐IL‐1β into its mature form, subsequently triggering a cascade of reactions that activate NLRP3 [31]. Grg3 can effectively ameliorate inflammatory damage in colitis by targeting the NLRP3 inflammasome pathway. Notably, research indicates a three‐ to fourfold elevation in NLRP3 expression within psoriatic lesions compared to healthy skin, alongside upregulated levels of IL‐1β and caspase‐1 [32]. In our study, we observed an increased expression of NLRP3 inflammasome‐related proteins compared to the normal group, which aligns with previous research findings. Significant reductions were observed in the levels of NLRP3, ASC, caspase‐1, and IL‐1β following Grg3 treatment, particularly the expression of IL‐1β. This reduction indicates that Grg3 exerts a positive inhibitory effect on the NLRP3 inflammasome. In conclusion, our research indicates that Grg3 can effectively improve psoriasis‐like dermatitis, and its effect is related to the inhibition of NLRP3 inflammasome activity. These preliminary findings suggest that inhibiting the NLRP3 inflammasome may be one of the potential mechanisms by which Grg3 exerts its protective effect.

Our data show that Grg3 concurrently suppressed NF‐κB activation and NLRP3 inflammasome signaling. Although the precise mechanistic link remains to be fully elucidated, it is plausible that the inhibition of NF‐κB, a master regulator of pro‐inflammatory gene expression, leads to reduced transcription of NLRP3 and IL‐1β, thereby priming the inflammasome less effectively. Future studies employing specific pathway inhibitors or Western blotting experiments are warranted to definitively establish the causal relationship and order of events between these pathways in the context of Grg3 treatment.

A key limitation is the restricted sample size, which compromises statistical robustness in discerning group disparities. While multiple trends corroborate earlier reports, select data failed to attain statistical significance. Future replication studies employing expanded cohorts are therefore essential to verify the proposed insights. While the present study demonstrates the promising efficacy of Grg3, it has some limitations. Most importantly, the maximum tolerated dose (MTD) was not formally established following standardized guidelines (e.g., OECD 420). This limits the precise definition of its therapeutic window at this stage. However, the absence of overt toxicity in our repeated‐dose study at efficacious levels is encouraging. As an immediate next step, a GLP‐compliant acute oral toxicity study following the OECD 420 fixed dose procedure will be conducted to determine the MTD and no‐observed‐adverse‐effect‐level. This data, together with chronic toxicity, genotoxicity, and safety pharmacology studies, will be critical for translating Grg3 into a viable clinical candidate for psoriasis. Although the known pharmacokinetics of Grg3 have been described in previous literature [33], accurate calculation of EC50 may also be required in future studies. More dose points should be set to accurately model the dose‐response relationship.

## 5 Conclusion

This study utilized a psoriasis‐like mouse model to preliminarily explore the therapeutic potential of Grg3 and its related mechanisms. The results indicated that Grg3 treatment could significantly alleviate psoriasis‐like skin lesions induced by IMQ and reduce the expression levels of key inflammatory factors (such as IL‐6, TNF‐α, IL‐17, IL‐1β). Mechanically, we speculate that Grg3 may interfere with the activation of the NF‐κB signaling pathway by inhibiting upstream factors such as IL‐6 and TNF‐α. The inhibition of this pathway may lead to a reduction in the production of downstream cytokines such as IL‐17 and IL‐1β. In addition, the decrease in IL‐1β levels may also indirectly affect the activation of NLRP3 inflammasomes, thereby interrupting the above pro‐inflammatory cascade reaction and ultimately alleviating psoriasis‐like inflammation. Meanwhile, Grg3 treatment also helps restore the immune balance of Th17/Treg cells. In conclusion, this study has initially revealed the efficacy and potential mechanism of Grg3 in alleviating psoriasis‐like dermatitis, providing a preliminary scientific basis for its use as a candidate drug for psoriasis treatment. However, its deeper target sites and precise mechanisms still need to be further explored.

## Author Contributions

Liyun Gao and Qingge Xie designed the study. Liyun Gao, Zhaoli Zhou, and Zhen Yue were responsible for data collection. Liyun Gao, Wanlu Zhang, and Huiya Sun conducted the data analysis. Liyun Gao drafted the manuscript, which was subsequently revised by Congjun Jiang. All authors reviewed the final version of the manuscript and approved its submission.

## Conflicts of Interest

The authors declare no conflicts of interest.
